# Supplementary material for: Calsequestrin localization at RyR2 clusters enables calcium wave propagation in ventricular myocytes
Source: PLoS Comput Biol. 2026 Oct 26;22(10):e1014449. doi: 10.1371/journal.pcbi.1014449 (PMC13754233; doi:10.1371/journal.pcbi.1014449)
Supplement: Supplementary file 1 [file pcbi.1014449.s001.pdf]

Supplementary Material: Calsequestrin localization at RyR2 clusters enables calcium wave propagation in ventricular myocytes

D. Conesa,[1] B. Echebarria,[2] L. Hove-Madsen,[3,4,5] Y. Shiferaw,[6] and E. Alvarez-Lacalle[2]
[1)] *Department of Physics, Universitat Politècnica de Catalunya - BarcelonaTech, Barcelona, Spain*
[2)] *Department of Physics and Institute for Research and Innovation in Health, Universitat Politècnica de Catalunya - BarcelonaTech, Barcelona, Spain*
[3)] *Institut de Recerca de Sant Pau, Hospital de la Santa Creu i Sant Pau, Barcelona, Spain*
[4)] *Instituto de Investigaciones Biomédicas de Barcelona (IIBB-CSIC), Barcelona, Spain*
[5)] *CIBER de Enfermedades Cardiovasculares (CIBERCV), Instituto de Salud Carlos III, Madrid, Spain*
[6)] *Physics Department, California State University Northridge, California, USA*

(*Electronic mail: enric.alvarez@upc.edu)

(Dated: 9 October 2026)

I. LIST OF CONTENTS

- Dynamical equations of the model.
- Numerical scheme and typical integration times.
- Intermediate levels of RyR2-CASQ2 co-localization.
- List of in-silico cell evolution videos.

II. DYNAMICAL EQUATIONS OF THE MODEL

A. Voxels

We define a $L_N \times W_N \times H_N$ voxel matrix. Each voxel is 100 nm $\times$ 100 nm $\times$ 100 nm. A large cell consists of $L_N = 1900, W_N = 300, H_N = 150$ ($190 \times 30 \times 15$ μm^3). Most of the simulations are done in a smaller matrix representing a thin central slice. We mostly use $L_N = 1900, W_N = 300, H_N = 9$ and then check that key results are recovered in the whole cell. A complete analysis in the full cell is computationally prohibitive.

B. Voxel compartments: cytosol and SR

In each voxel, indexed by r, we define a cytosolic compartment and an SR compartment. The respective ratios of the total volume are v_i and v_{SR}.

In the cytosolic compartment of all voxels, we define the free concentration of calcium c_i^r, the calcium concentration bound to each of the buffers in the cytosol $c_{b_j}^r$, where j runs from 1 to 5 since we consider five buffers: Troponin-C ($j = 1$), Calmodulin (CaM) ($j = 2$), SR-binding sites ($j = 3$), SL-binding sites ($j = 4$) and Fluo-4 ($j = 5$). Then we can define the total calcium bound in the cytosol in voxel r as $c_b^r = \sum_{j=1}^{5} c_{b_j}^r$ and the total calcium in the cytosol as the sum of free and bound calcium $C_T^r = c_i^r + c_b^r$.

In the SR, we define the free SR calcium concentration c_{SR}^r and the amount of calcium bound to Calsequestrin c_{bSR}^r. The total amount of calcium in the SR in voxel r is: $C_{SRTOT}^r = c_{SR}^r + c_{bSR}^r$. In the manuscript, we investigate different spatial distributions of CASQ2. The physiological (base) model has Calsequestrin only in the voxel with RyR2.

C. Voxel dynamics

The calcium cycling dynamics of a given voxel at position r are the same in all the models used in the manuscript. They read:

$$\frac{dC_T^r}{dt} = J_{\text{ext}}^r + J_{\text{rel}}^r - J_{\text{SERCA}}^r - J_{\text{NCX}}^r + D_i \nabla^2 c_i \quad (1)$$

$$\frac{dC_{\text{SRTOT}}^r}{dt} = \frac{v_i}{v_{\text{SR}}}(J_{\text{SERCA}}^r - J_{\text{rel}}^r) + D_{\text{SR}} \nabla^2 c_{\text{SR}} \quad (2)$$

$$\frac{dc_b^r}{dt} = \sum_{j=1}^{5} k_j^+ \, c_i^r \left(B_j - c_{b_j}^r\right) - k_j^- \, c_{b_j}^r \quad (3)$$

$$c_i^r = C_T^r - c_b^r \quad (4)$$

$$C_{\text{SRTOT}}^r = c_{SR}^r + c_{bSR}^r = c_{SR}^r + c_{SR}^r \frac{B_{\text{CASQ2}}^r}{K_{\text{CASQ2}} + c_{SR}^r} \quad (5)$$

where B_j, k_j^+, and k_j^- indicate the total amount of buffer j and the on and off rate of each buffer. Notice that in the cytosol, all voxels have the same amount of buffer. Meanwhile, we use instantaneous equilibrium in the SR for the only buffer present, CASQ2. The key feature of our model is that the Calsequestrin content depends on the voxel; therefore, B_{CASQ2}^r depends on the voxel. In the model where CASQ2 is colocalized with the RyR2, Calsequestrin is zero in most voxels. Only in those voxels with RyR2 clusters is CASQ2 present.

Finally, the diffusion term uses no-flux boundary conditions at the border.

In those voxels where RyR2 and CASQ2 are present, we assume fast buffering kinetics and obtain the free Ca^{2+} concentration in the SR c_{SR} from the total SR Ca^{2+} concentration

$c_{\mathrm{SR}}^{\mathrm{TOT}}$ solving a quadratic equation as described in[1]:

$$c_{\mathrm{SR}} = \frac{-b+\sqrt{b^2+4K_{\mathrm{CASQ2}}c_{\mathrm{SR}}^{\mathrm{TOT}}}}{2} \tag{6}$$

where $b = K_{\mathrm{CASQ2}} + B_{\mathrm{CASQ2}} - c_{\mathrm{SR}}^{\mathrm{TOT}}$, and B_{CASQ2} is the total buffering capacity of CASQ2, and K_{CASQ2} the dissociation constant of the Ca-CASQ2 complex.

D. Currents

The equations for the different currents are the following:

J_{ext}^r is zero everywhere except when an external excitation is introduced to generate a wave. This is done by introducing a constant current at a specific location r during 40 ms. The typical coordinate used is close to the upper left corner of the center plane. This is the 30th voxel in length, the 24th voxel in width, and the 5th voxel in height.

The current from the SERCA pump is present in all voxels and is defined as:

$$J_{SERCA}^r = g_{up}\frac{(\frac{c_i^r}{K_i})^H - (\frac{c_{SR}^r}{K_{SR}})^H}{1+(\frac{c_i^r}{K_i})^H + (\frac{c_{SR}^r}{K_{SR}})^H} \tag{7}$$

On the other hand, the exchanger current is only present in voxels where a Na-Ca exchanger is present. We can mathematically state that the strength of the current g_{NCX}^r depends on the voxel, being zero when NCX is not present. The current reads then:

$$J_{NCX}^r = g_{NCX}^r \frac{e^{-0.65z}}{1+k_{sat}e^{-0.65z}} \frac{c_i^r - e^z c_o(\frac{Na_i}{Na_o})^3}{1+(\frac{K_{NCX}}{c_i^r})^3} \tag{8}$$

Finally, the calcium released from the RyR is also voxel-dependent. Those voxels without RyR have zero conductivity and no release. This means that mathematically, the conductivity can be written as g_{rel}^r, where this conductivity is zero unless r indicates a voxel with RyR2, in which case it is g_{rel}. The calcium release also depends on the ratio of RyR in the open state. The dynamics and regulation of RyR fix this opening rate as described in the main manuscript. We provide here a summary of the equations:

$$J_{rel}^r = g_{rel}^r O_{RyR}^r (c_{SR}^r - c_i^r) \tag{9}$$

$$\frac{dO_{RyR}^r}{dt} = k_+^r(R^r - O_{RyR}^r) - k^- O_{RyR}^r \tag{10}$$

$$\frac{dR^r}{dt} = \frac{1}{\tau_{rec}}(1-R^r) - k_{in}^r(R^r - O_{RyR}^r) \tag{11}$$

$$k_+^r = K_p \cdot \frac{\left(\frac{c_i^r}{c_i^*}\right)^{H_{k+}}}{1+\left(\frac{c_i^r}{c_i^*}\right)^{H_{k+}}} \cdot [\alpha + (1-\alpha)\cdot o_{\mathrm{LCA}}^r] \tag{12}$$

$$k_{\mathrm{in}} = k_{\mathrm{in}}^* \cdot o_{\mathrm{CaM}}^r \cdot [\beta + (1-\beta)\cdot o_{\mathrm{LCA}}^r] \tag{13}$$

$$\frac{do_{\mathrm{LCA}}^r}{dt} = -k_{LCa}^+ (c_{SR}^r)^H (1-o_{\mathrm{LCA}}^r) - k_{LCA}^- o_{\mathrm{LCA}}^r \tag{14}$$

$$\frac{do_{\mathrm{CaM}}^r}{dt} = K_{\mathrm{IN}}^+ c_{b_2}^r (1-o_{\mathrm{CaM}}^r) - K_{\mathrm{IN}}^- o_{\mathrm{CaM}}^r \tag{15}$$

where $k_{\mathrm{LCA}}^+ = k_{\mathrm{LCA}}$ and $k_{\mathrm{LCA}}^- = k_{\mathrm{LCA}} K_{SR}^H$. It is important to recall that $c_{b_2}^r$ is the calcium bound to calmodulin $[Ca \cdot \mathrm{CaM}]$.

The initial condition sets all buffers and internal states of the RyR2 at equilibrium at a given calcium concentration in all voxels. Given this, once the free calcium in the cytosol and in the SR are selected, the total amount of calcium present initially in the cell is fixed, together with all the initial RyR2 occupation states. For example, if we select an initial free cytosolic calcium concentration of $c_i(t=0)$ at 200 nM, then, at equilibrium, the calcium bound to the buffers is 40.6 μM. Similarly, if we set the initial free calcium concentration in the SR $c_{SR}(t=0)$ at 1 mM, then the total amount of calcium in the SR includes this free calcium plus the calcium bound to CASQ2 in the voxels with RyR2. The calcium bound to CASQ2 is 1.468 mM on average in the SR, yielding a total calcium concentration in the SR of $c_{SR}^{TOT} = 2.468$ mM. Using the volume ratio of our model (95% cytosol, 5% SR), these numbers yield a $C_{TOT} = c_T f_i + c_{SR}^{TOT} f_{SR} = 162\ \mu mol/l_{cell}$. The total calcium concentration is the governing parameter that determines the system's evolution.

III. MODEL IMPLEMENTATION

The numerical implementation has been carried out in MATLAB and FORTRAN to double-check the results, using a 3D matrix to define the spatial voxel division of the cell. The basic structure of the code starts with an initialization process and then uses a hybrid implementation to obtain the time update with the diffusion term. We proceed to explain each one of these parts.

A. Initialization

The initialization process is primarily structured in two parts, in addition to the initial definition of the parameters. The first part involves creating the 3D matrices for the different spatial variables (those with a superscript r in the previous

section), including those representing the information about which voxels contain a RyR2 cluster and which contain NCX. We use a matrix R^r_{array} of 0s and 1s to indicate the presence of RyR2. 0 indicates no RyR2 in this voxel, while 1 indicates its presence. Similarly, another matrix NCX^r_{array} is created to represent the location of NCX. By element-wise multiplying this matrix with the release or NCX current matrices, we can set the current to zero in the voxels where the respective channels are absent.

The second part of the initialization involves setting the total amount of calcium in the cell to a desired value. To do this, we set a uniform free calcium concentration in the cytosol and a free calcium concentration in the SR. We always set a combination that gives a round value to the total calcium concentration in the cell once we have set the buffers in equilibrium so that $c_{b_j}(t=0) = c_i(t=0)\frac{B_j}{K_j+c_i(t=0)}$. We apply the same process for Calsequestrin in the SR.

B. Numerical implementation

1. General structure

We use a time step of $\Delta t = 0.02$ ms to guarantee stability and accuracy with the use a hybrid propagation scheme described below. We have checked that the results are the same for $\Delta t = 0.001$ using a standard Euler method.

The first step is to update the occupation steps Eq. 14 and 15. Notably, the first one is implicitly given that the time rates can be relatively close to our time step. If we define $o^r_{LCA}(t)$ as the matrix which contains the occupation rates of the RyR at time step t, we update it according to:

$$\Delta h = \Delta t \cdot [k^+_{LCA}(c^r_{SR}(t-\Delta t)^H + k^-_{LCA}] \tag{16}$$

$$o^r_{LCA}(t) = \frac{o^r_{LCA}(t-\Delta t) + \Delta t \cdot k^+ c^r_{SR}(t-\Delta t)^H}{1+\Delta h} \tag{17}$$

After this update, we compute the matrix rates in Eq. 12 and 13 and proceed to update the states of the RyR2. This is, we solve Eqs 10 and 11 using a first-order Euler explicit method.

Once the states are updated, we compute the SERCA current Eq. 7, the NCX current Eq. 8 and the current from the RyR in Eq. 9. In the last two, we use the corresponding binary matrix R^r_{array} and NCX^r_{array} to multiply the terms to specify the voxels where these currents exist. For example, the release current at time t is implemented as

$$J^r_{rel}(t) = R^r_{array} \cdot g_{rel} O^r_{RyR}(t) \cdot [c^r_{SR}(t-\Delta t) - c^r_i(t-\Delta t)] \tag{18}$$

where $O^r_{RyR}(t)$ has already been updated. For SERCA, since it is in all voxels, it would read:

$$J^r_{SERCA}(t) = g_{up}\frac{(\frac{c^r_i(t-\Delta t)}{K_i})^H - (\frac{c^r_{SR}(t-\Delta t)}{K_{SR}})^H}{1+(\frac{c^r_i(t-\Delta t)}{K_i})^H + (\frac{c^r_{SR}(t-\Delta t)}{K_{SR}})^H} \tag{19}$$

The next step is to compute the flux of the diffusion term in both the SR and the cytosol using the previous value of free calcium in the SR and the cytosol. This will be used later. To do this, we use the six nearest neighbors. For the SR, we compute the diffusion flux at each voxel$D_{SR}\nabla^2 c_{SR} = J^r_{SRDIFF} = J^{xyz}_{SRDIFF}$ as

$$J^{xyz}_{SRDIFF}(t) = \frac{D_{SR}}{\Delta x^2}[c^{x+1,y,z}_{SR}(t-\Delta t) + c^{x-1,y,z}_{SR}(t-\Delta t) \tag{20}$$

$$+ c^{x,y+1,z}_{SR}(t-\Delta t) + c^{x,y-1,z}_{SR}(t-\Delta t)+ \tag{21}$$

$$c^{x,y,z+1}_{SR}(t-\Delta t) + c^{x,y,z-1}_{SR}(t-\Delta t) - 6c^{x,y,z}_{SR}(t-\Delta t)], \tag{22}$$

plus no-flux boundary conditions. The same approach is used to compute $D_i\nabla^2 c_i = J^r_{CYTDIFF}$.

Once we have all the currents, we compute the change in the total amount of calcium in the SR using a fully explicit method. This reads:

$$J^r_{SRTOT}(t) = J^r_{\text{SERCA}}(t) - J^r_{\text{rel}}(t) + J^r_{SRDIFF}(t) \tag{23}$$

$$C_{SRTOT}(t) = C_{SRTOT}(t-\Delta t) + \Delta t\frac{v_i}{v_{\text{SR}}}J_{SRTOT}(t) \tag{24}$$

Then, from C_{SRTOT} we compute the free SR calcium solving the quadratic equation Eq. 26.

$$b^r = K_{\text{CASQ2}} + B^r_{\text{CASQ2}} - C^r_{\text{SRTOT}} \tag{25}$$

$$c^r_{SR} = \frac{-b^r + \sqrt{(b^r)^2 + 4K_{\text{CASQ2}}C^r_{SRTOT}}}{2} \tag{26}$$

Finally, the last step is to update the free calcium in the cytosol and the calcium attached to the buffers. We use a hybrid explicit-implicit split of the diffusion term to improve accuracy and stability with a time step large enough, explained in the following subsection.

2. Hybrid explicit-implicit split method to compute calcium concentration in the cytosol

We use an explicit-implicit split but with a hybrid time step for the free and total concentrations to get very good accuracy and stability for a large Δt. The basic goal is to implement Eqs. 1, 3, and 4 numerically efficient but also in a way that conserves exactly the total calcium mass of the problem. To this aim, we use the hybrid explicit-implicit split. First, we use the explicit computation of the diffusion term and the release, NCX, SERCA, and external current to update the total calcium in the cell.

$$J^r_{cyt}(t) = J^r_{ext}(t) + J^r_{rel}(t) - J^r_{SERCA}(t) - J^r_{NCX}(t) \tag{27}$$

$$C^r_T(t) = C^r_T(t-\Delta t) + \Delta t[J^r_{cyt}(t) + J^r_{CYTDIFF}(t)] \tag{28}$$

Using explicit methods, we also update each calcium bound to each buffer.

$$J_{b_j}(t) = k_j^+ c_i^r(t-\Delta t)[B_j - c_{b_j}^r(t-\Delta t)] - k_j^- c_{b_j}^r(t-\Delta t) \quad (29)$$

$$c_{b_j}^r(t) = c_{b_j}^r(t-\Delta t) + \Delta t J_{b_j}(t) \quad (30)$$

Summing all, we can compute the total calcium bound to buffers in the cytosol:

$$c_b^r(t) = \sum_{j=1}^{5} c_{b_j}(t) \quad (31)$$

With these equations, total calcium in the cytosol is exactly conserved. Now, from these values, we use a hybrid half-step method with an explicit-implicit split. The algorithm to obtain $c_i^r(t)$ is the following:

$$c_i^r(t-\Delta t/2) = C_T^r(t) - c_b^r(t) \quad (32)$$

$$\Delta D_c = 6\Delta t D_i/\Delta x^2 \quad (33)$$

$$c_i^r(t) = \frac{c_i^r(t-\Delta t/2) + \Delta D_c c_i^r(t-\Delta t)}{1+\Delta D_c} \quad (34)$$

These equations implement an explicit-implicit split method to account for the diffusion term without affecting the total calcium level, which is fixed in the total amount of calcium. The code is stable and pretty accurate compared with simulations, which has a much smaller time step but uses a fully explicit method.

INTERMEDIATE LEVELS OF RYR2-CASQ2 CO-LOCALIZATION

In the main manuscript, we considered two limiting cases of RyR2-CASQ2 spatial organization: full co-localization, in which all CASQ2 is located in the same voxels as RyR2 clusters, and uniform CASQ2 distribution, in which CASQ2 is distributed throughout the SR. We showed that co-localization of CASQ2 with RyR2 is necessary for sustained calcium wave propagation. Here, we investigate intermediate levels of co-localization to determine the minimum degree of CASQ2 localization required to sustain wave propagation in our model.

To vary the degree of co-localization, we distributed the total CASQ2 content between the voxels containing RyR2 clusters and the remaining voxels. We considered co-localization levels of 80 %, 60 %, and 40 %, with the remaining 20 %, 40 %, and 60 % of the total CASQ2 content, respectively, distributed uniformly throughout the SR. Thus, for example, at 80 % co-localization, 80 % of the total CASQ2 is localized to voxels containing RyR2, while the remaining 20 % is uniformly distributed across the other voxels.

Figure A shows the resulting calcium wave dynamics for a total calcium load of 180 μmol/$l_{\rm cell}$, comparable to the conditions analyzed in the main manuscript. At 80 % and 60 % co-localization, the initial wave propagates throughout the cell and remains sustained for the duration of the simulation. However, reducing co-localization from 80 % to 60 % results in a lower propagation velocity and a slightly smaller wave width. In contrast, at 40 % co-localization, the wave fails to propagate along the cell. These results indicate that, within our model, more than half of the total CASQ2 content needs to be co-localized with RyR2 clusters for sustained wave propagation.

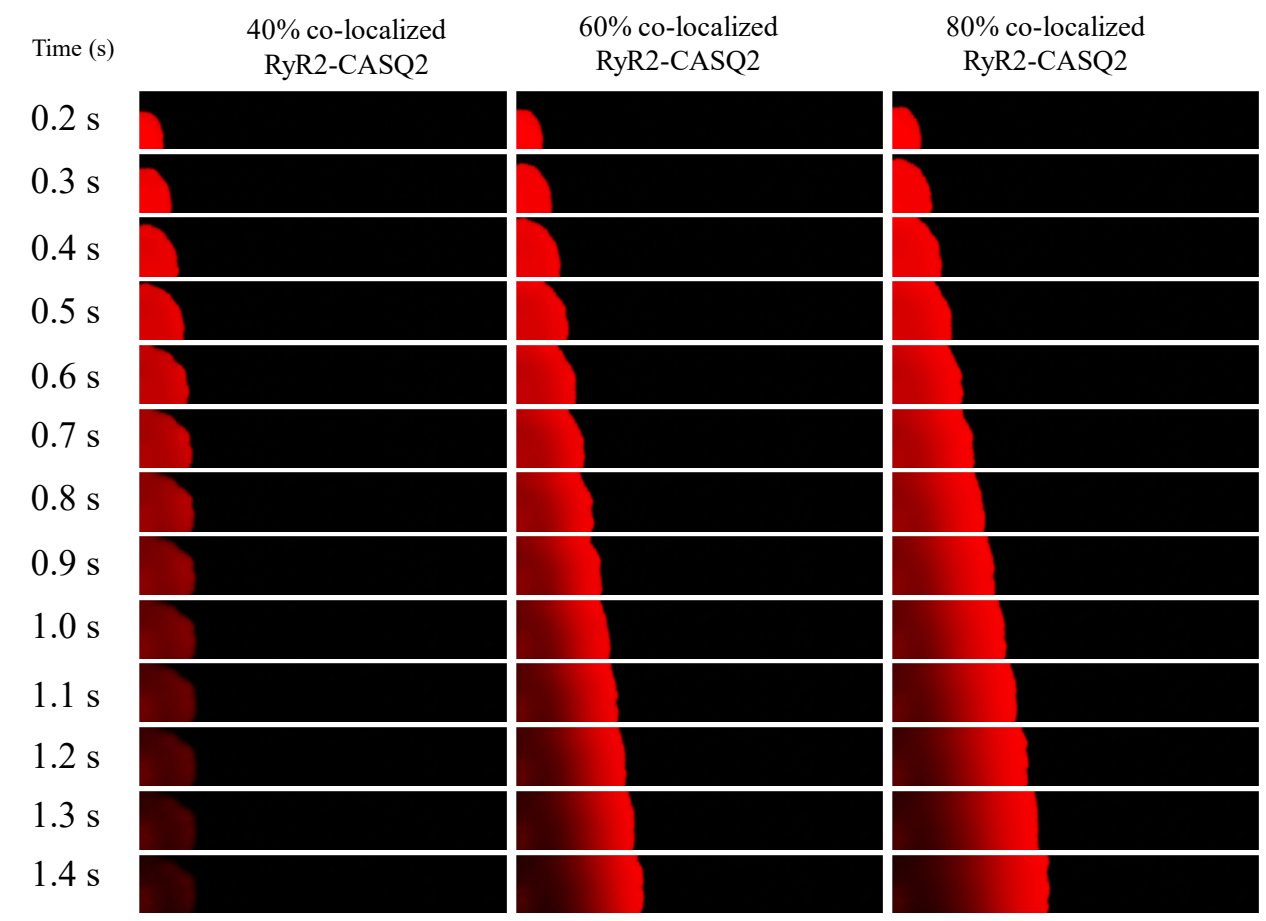

FIG. A. **Calcium wave propagation at different levels of RyR2-CASQ2 co-localization.** From left to right, evolution of calcium fluorescence during the first 1.4s of the simulation for 40 %, 60%, and 80 % RyR2-CASQ2 co-localization at a total calcium load of 180μmol/$l_{\rm cell}$. At 80 % and 60 % co-localization, the calcium wave propagates throughout the cell, with 60 % co-localization resulting in a lower propagation velocity and a slightly smaller wave width. At 40 % co-localization, propagation fails.

Interestingly, the results obtained with 80 % co-localization are very similar to those obtained with full co-localization, indicating that the wave dynamics are relatively insensitive to moderate reductions in CASQ2 localization. Together, these results show that the main conclusions of the manuscript are robust to intermediate levels of RyR2-CASQ2 co-localization, with sustained wave propagation maintained for co-localization levels between approximately 60 % and full co-localization.

[1]D. Conesa, B. Echebarria, A. Peñaranda, I. R. Cantalapiedra, Y. Shiferaw, and E. Alvarez-Lacalle, "Two-variable nullcline analysis of ionic general equilibrium predicts calcium homeostasis in ventricular myocytes," PLoS Computational Biology **16**, e1007572 (2020).

VIDEO RESOURCES AND MODEL DESCRIPTIONS

Below is a comprehensive list of the simulation videos generated from our base model and its variations, which are available on GitHub. We provide a brief description of what can be seen in the videos (see Table D for the complete list of files).

Definitions: We define an "open state" (or domains with elevated cytosolic calcium) as an area where cytosolic $[Ca^{2+}]$ remains high, the sarcoplasmic reticulum (SR) remains persistently depleted, and a fraction of the RyR2 receptors remain open for a prolonged period.

1. Physiological (base) Model

The naming convention for the base model videos is `VideoBLoad###.mp4`, where `###` represents the total calcium concentration ($[Ca^{2+}]_{total}$) in the cell.

2. NCX Blocked (Knockout)

These videos illustrate the scenario where NCX is blocked. The naming convention is `VideoNCXKOLoad###.mp4`, where `###` stands for the total calcium concentration. *(Note: File names include an extra 'B' after NCX).*

3. NCX Upregulated

These videos demonstrate the system when NCX is upregulated by a factor of 16. The naming convention is `VideoNCXURLoad###.mp4`. *(Note: File names include an extra 'B' after NCX).*

4. Uniform CASQ2

These videos show the case with uniform CASQ2 and NCX active at the standard extrusion rate (identical to the base model). The naming convention is `VideoCASQUNIFORMLoad###.mp4`, where `###` stands for the total load.

5. Intermediate RyR2-CASQ2 colocalization

These videos show the case with intemedaite values of RyR2-CASQ2 co-localizaton and NCX active at the standard extrusion rate (identical to the base model) and at an initial total calcium load of 180 μmol/l_{cyt}. The naming convention is `VideoCSQ##Load180.mp4`, where `##` stands for the perfentatge of CASQ2 co-localized with RyR2.

Table A. **Buffer parameters at room temperature**

Symbol	Meaning	Value
B_{TnC}	Troponin-C conc.	$70\,\mu$M
k^+_{TnC}	TnC binding	$0.0131\,\mu M^{-1} ms^{-1}$
k^-_{TnC}	TnC unbinding	$0.00784\,ms^{-1}$
B_{CaM}	Calmodulin conc.	$25\,\mu$M
k^+_{CaM}	CaM binding	$0.012\,\mu M^{-1} ms^{-1}$
k^-_{CaM}	CaM unbinding	$0.072\,ms^{-1}$
B_{bSR}	SR sites conc.	$20\,\mu$M
k^+_{bSR}	SR binding	$0.04\,\mu M^{-1} ms^{-1}$
k^-_{bSR}	SR unbinding	$0.024\,ms^{-1}$
B_{FL}	Rhod-2 conc.	$50\,\mu$M
k^+_{RH}	Rhod-2 binding	$0.04\,\mu M^{-1} ms^{-1}$
k^-_{RH}	Rhod-2 unbinding	$0.028\,ms^{-1}$
B_{slh}	SL sites conc.	$15\,\mu$M
k^+_{slh}	SL binding	$0.04\,\mu M^{-1} ms^{-1}$
k^-_{slh}	SL unbinding	$0.012\,ms^{-1}$
$CASQ2^{TOT}$	CASQ2 (SR)	3.23 mM
K_{CASQ2}	Dissociation const.	1.2 mM

Table B. **Structural and SERCA parameters at room temperature**

Symbol	Meaning	Value
f_i	Cytosolic fraction	0.95
f_{SR}	SR fraction	0.05
D_i	Cytosol diffusion	$0.32\,\mu m^2\,ms^{-1}$
D_{SR}	SR diffusion	$0.032\,\mu m^2\,ms^{-1}$
g_{up}	SERCA max uptake	$0.25\,\mu M\,ms^{-1}$
K_i	Ca^{2+} half-sat.	$0.123\,\mu$M
K_{SRCa}	SR half-sat.	$1300\,\mu$M
H	Hill coeff. (SERCA)	2

Table C. **RyR2 and NCX parameters at room temperature**

Symbol	Meaning	Value
g_{rel}	Release strength	$3.2\,ms^{-1}$
k^-	Closing rate	$0.04\,ms^{-1}$
τ_{rec}	Recovery time	1000 ms
K_p	Opening rate factor	$0.2\,ms^{-1}$
k^*_{in}	Inactivation factor	$0.0048\,ms^{-1}$
c^*_i	Cytosolic Ca^{2+} (half)	$5\,\mu$M
H_{k^+}	Hill coeff. (cyto)	3
K_{SR}	SR affinity	$700\,\mu$M
α	Gate reduction	$1/30$
β	Gate increase	31
H_b	Hill coeff. (SR)	5
k_{LCa}	Luminal binding rate	$0.08\,ms^{-1}$
K^+_{IN}	CaM binding	$0.004\,\mu M^{-1} ms^{-1}$
K^-_{IN}	CaM unbinding	$0.04\,ms^{-1}$
g_{NCX}	NCX rate	$0.06\,ms^{-1}$
k_{sat}	Saturation constant	0.27
K_{NCX}	Inactivation	$0.25\,\mu$M
η	Voltage factor	0.65
c_o	Extracellular Ca^{2+}	1.8 mM
Na_o	Extracellular Na^+	145 mM
Na_i	Intracellular Na^+	3 mM

Table D. Simulation videos available on GitHub https://github.com/ConesaJR/CASQ2_RyR2_waves and their corresponding dynamics. One second of simulation corresponds to two seconds in movie time

1. Physiological (base) Model

File Name	Description
VideoBLoad170.mp4	Triggered activity advances a few Z-planes and stops.
VideoBLoad175.mp4	Triggered single wave.
VideoBLoad180.mp4	Triggered single wave.
VideoBLoad190.mp4	Triggered wave and global activation and then a return to the resting state.
VideoBLoad200.mp4	Triggered wave and global activation, followed by a spontaneous waves, and then a return to the resting state.

2. NCX Blocked (Knockout) Videos

File Name	Description
VideoNCXBKOLoad180.mp4	Triggered single wave. Returns to a homogeneous state with the same total calcium as the initial state.
VideoNCXBKOLoad190.mp4	Global activity that does not return to the homogenous resting state.

3. NCX Upregulated Videos

File Name	Description
VideoNCXBURLoad180.mp4	Triggered single wave interrupted.
VideoNCXBURLoad190.mp4	Triggered single wave.

4. Uniform CASQ2 Videos

File Name	Description
VideoCASQUNIFORMLoad180.mp4	Non-persistent wave.
VideoCASQUNIFORMLoad190.mp4	Global activation leads to the formation of domains that remain active on the order of minutes, eventually disappearing.

5. Intermediate CASQ2-RyR2 co-localization Videos

File Name	Description
VideoCSQ40Load180.mp4	No propagation of waves with 40 % of CASQ2 co-localized with RyR2
VideoCSQ60Load180.mp4	Propagation of waves with 60 % of CASQ2 co-localized with RyR2 but with lower velocity that full co-localization.
VideoCSQ80Load180.mp4	Propagation of waves with 80 % of CASQ2 co-localized with RyR2, very similar to full co-localziation.
