# Supplementary material for: Calsequestrin localization at RyR2 clusters enables calcium wave propagation in ventricular myocytes
Source: PLoS Comput Biol. 2026 Oct 26;22(10):e1014449. doi: 10.1371/journal.pcbi.1014449 (PMC13754233; doi:10.1371/journal.pcbi.1014449)
Supplement: Supplementary file 2 [file pcbi.1014449.s003.pdf]

Report on “Calsequestrin localization at RyR2 clusters enables calcium wave propagation in ventricular myocytes”

This manuscript presents a three-dimensional reaction–diffusion model of calcium cycling in mouse ventricular myocytes, with a spatial discretization of 100 nm. The model includes cytosolic and SR calcium diffusion, RyR2-mediated release, SERCA uptake, NCX extrusion, cytosolic buffering, and spatially varying CASQ2 buffering in the SR. The main result is that calcium waves propagate robustly when CASQ2 is concentrated near RyR2 clusters, but fail when the same total amount of CASQ2 is distributed uniformly throughout the SR. The proposed mechanism is that localized CASQ2 provides a local luminal calcium reservoir that sustains SR calcium concentration and release flux at the wavefront. The authors also investigate the role of NCX and conclude that NCX is not required for wave termination at moderate calcium loads, but becomes important at higher loads by reducing the total cellular calcium content. The model reproduces experimentally observed wave velocities and is compared with calcium-wave recordings from isolated ventricular myocytes.

The paper is well written and easy to follow, and the results are interesting and important. The model is well formulated and appears to generate physiologically reasonable results.

Major comments

1. Given the complexity of the model, I think the manuscript would benefit from a schematic overview showing the different compartments, the variables defined in each compartment, the fluxes between them, and the buffers involved. At present, some of this information is spread between the main text and the Supplementary Material, which makes the model somewhat harder to parse than necessary.
2. The model treats CASQ2 buffering in the SR through an instantaneous equilibrium relation rather than a separate kinetic equation. Since the central conclusion depends on local CASQ2 buffering sustaining SR calcium release, this approximation should be justified. In particular, the authors should clarify whether CASQ2 binding and unbinding are sufficiently fast relative to SR depletion and wave propagation, or show that the main result is robust when finite CASQ2 kinetics are included.
3. The final paragraph of the Methods section partly presents simulation outcomes, particularly the statements about calcium retention and the effects of NCX knockout. These points would fit better in the Results section, while the Methods should be restricted to describing how NCX activity was modified.
4. The term “nanometric model” may be misleading. Although the spatial discretization is 100 nm, the model is a reaction–diffusion formulation describing calcium dynamics over submicron and micrometre distances. The terminology should distinguish the present formulation from models intended to resolve nanometre-scale electrodiffusion, including electrical forces and Debye-layer effects, using the PNP equations; see, for example, [1–3].
5. The study emphasizes that the spatial localization of buffering can strongly affect wave propagation. However, all cytosolic buffers are assumed to be uniformly distributed. It would be useful to comment on whether non-uniform localization of cytosolic buffers near RyR2, SERCA, or NCX could also influence the results. Mathematically, it appears plausible that high buffer concentrations in the vicinity of these transport proteins could considerably affect the local calcium dynamics.
6. The discussion occasionally presents CASQ2 co-localization as “solving” the source-strength problem required for wave propagation. This wording seems physiologically

misleading, since the waves considered here are spontaneous and potentially arrhythmogenic. CASQ2 localization does not solve a requirement of normal excitation; rather, in the model it provides sufficient local source strength for pathological calcium waves to propagate. This distinction should be made clearer.

7. The discussion could also mention a broader modelling implication of the results. The marked difference between localized and uniformly distributed CASQ2 suggests that spatially averaged representations of proteins may be inadequate when local concentrations near channels or transporters determine the dynamics. More generally, the results caution against replacing heterogeneous protein distributions by uniform effective concentrations in spatially resolved models.
8. Several previous studies have considered spatially resolved modelling of calcium sparks and waves in ventricular myocytes, including the initiation and propagation of spontaneous waves, the role of microscopic dyadic function, and the influence of subcellular geometry and protein localization [4–6]. These studies appear relevant to the present work and should be considered in positioning the model and its contribution relative to the existing literature.

Minor comments

1. Reference [2] is incomplete.
2. Reference [20] has an incorrect author list; Lederer is not the first author.
3. The cell size is specified as follows: “The cell is represented as a 3D grid of voxels, each measuring 100 nm × 100 nm × 100 nm (see Figure 1). A typical full cell consists of 300 × 1900 × 150 voxels.” This corresponds to a volume of 85.5 pL, which appears unusually large for a mouse ventricular cardiomyocyte. Please clarify the relation between the rectangular computational domain and the physiological cell volume.
4. I found the statement “with calcium ions diffusing between neighboring voxels in both the SR and the cytosol” somewhat unclear. It may be read as implying that calcium diffuses freely between the SR and cytosol, which would be incorrect. The intended meaning is clear from the equations, but the text should be made unambiguous.
5. The statement “O_{RyR} is the fraction of RyR2 in the clusters that are in the open state” is also somewhat confusing. O_{RyR} appears to be a local variable representing the open probability of a channel, or equivalently the expected local fraction of open channels. Should equations (4) and (5) be interpreted locally, with separate values in each RyR2-containing voxel, or are the same values used throughout the cell?
6. The Data Availability statement indicates that the source code is available only from the corresponding author upon reasonable request. Since the conclusions depend on a substantial computational model, the full source code, parameter files, and instructions required to reproduce the simulations should be made directly available in a public repository.

References

[1] Lu et al. 2010. Poisson–Nernst–Planck equations for simulating biomolecular diffusion-reaction processes I: finite element solutions.

[2] Lu et al. 2022. Poisson–Nernst–Planck equations for simulating biomolecular diffusion-reaction processes II: size effects on ionic distributions and diffusion-reaction rates.

[3] Jæger et al. 2025. Electrodiffusion dynamics in the cardiomyocyte dyad at nano-scale resolution using the Poisson–Nernst–Planck (PNP) equations.

[4] Li et al. 2010. Computational modelling of the initiation and development of spontaneous intracellular Ca^{2+} waves in ventricular myocytes.

[5] Gaur et al.. 2011. Multiscale modeling of calcium cycling in cardiac ventricular myocyte: macroscopic consequences of microscopic dyadic function.

[6] Hake et al. 2012. Modelling cardiac calcium sparks in a three-dimensional reconstruction of a calcium release unit.
