# Supplementary material for: Calsequestrin localization at RyR2 clusters enables calcium wave propagation in ventricular myocytes
Source: PLoS Comput Biol. 2026 Oct 26;22(10):e1014449. doi: 10.1371/journal.pcbi.1014449 (PMC13754233; doi:10.1371/journal.pcbi.1014449)
Supplement: Supplementary file 4 [file pcbi.1014449.s004.docx]

Referee1. Report on “Calsequestrin localization at RyR2 clusters enables calcium wave propagation in ventricular myocytes”

This manuscript presents a three-dimensional reaction–diffusion model of calcium cycling in

mouse ventricular myocytes, with a spatial discretization of $100$ nm. The model includes

cytosolic and SR calcium diffusion, RyR2-mediated release, SERCA uptake, NCX extrusion,

cytosolic buffering, and spatially varying CASQ2 buffering in the SR. The main result is that

calcium waves propagate robustly when CASQ2 is concentrated near RyR2 clusters, but fail

when the same total amount of CASQ2 is distributed uniformly throughout the SR. The

proposed mechanism is that localized CASQ2 provides a local luminal calcium reservoir that

sustains SR calcium concentration and release flux at the wavefront. The authors also

investigate the role of NCX and conclude that NCX is not required for wave termination at

moderate calcium loads, but becomes important at higher loads by reducing the total cellular

calcium content. The model reproduces experimentally observed wave velocities and is

compared with calcium-wave recordings from isolated ventricular myocytes.

The paper is well written and easy to follow, and the results are interesting and important. The model is well formulated and appears to generate physiologically reasonable results.

Major comments

1. Given the complexity of the model, I think the manuscript would benefit from a schematic overview showing the different compartments, the variables defined in each compartment, the fluxes between them, and the buffers involved. At present, some of this information is spread between the main text and the Supplementary Material, which makes the model somewhat harder to parse than necessary.

We agree with the referee and we have now changed Fig. 1 which is now larger and more comprehensive in its details of the different compartments and fluxes with the addition of a new panel D.

2. The model treats CASQ2 buffering in the SR through an instantaneous equilibrium relation rather than a separate kinetic equation. Since the central conclusion depends on local CASQ2 buffering sustaining SR calcium release, this approximation should be justified. In particular, the authors should clarify whether CASQ2 binding and unbinding are sufficiently fast relative to SR depletion and wave propagation, or show that the main result is robust when finite CASQ2 kinetics are included.

Standard values in the literature (e.g. Shannon & Bers) consider the on and off rates of $\text{CASQ2--}\text{Ca}^{2+}$ binding as $k_{\text{on}}=100\text{ mM}^{-1}\text{ms}^{-1}$ and $k_{\text{off}}=65\text{ ms}^{-1}$. This means that the binding and unbinding of $\text{Ca}^{2+}$ to $\text{CASQ2}$ occur on sub-millisecond time scales and are, therefore, much faster than SR depletion or wave propagation. Under these conditions, assuming instantaneous buffer equilibrium is perfectly justified. Including the explicit kinetics of $\text{CASQ2--}\text{Ca}^{2+}$ binding would require a much smaller numerical time step, significantly increasing computation time without altering the wave dynamics.

3. The final paragraph of the Methods section partly presents simulation outcomes, particularly the statements about calcium retention and the effects of NCX knockout. These points would fit better in the Results section, while the Methods should be restricted to describing how NCX activity was modified.

We agree- We have rewritten this part following the indication of the referee.

4. The term “nanometric model” may be misleading. Although the spatial discretization is $100$ nm, the model is a reaction–diffusion formulation describing calcium dynamics over submicron and micrometre distances. The terminology should distinguish the present formulation from models intended to resolve nanometre-scale electrodiffusion, including electrical forces and Debye-layer effects, using the PNP equations; see, for example, [1–3].

We agree with the referee. Now we refer to the model as subcellular and submicrometer model. We now acknowledge this distinction in the methodology, when we present our model.

5. The study emphasizes that the spatial localization of buffering can strongly affect wave propagation. However, all cytosolic buffers are assumed to be uniformly distributed. It would be useful to comment on whether non-uniform localization of cytosolic buffers near RyR2, SERCA, or NCX could also influence the results. Mathematically, it appears plausible that high buffer concentrations in the vicinity of these transport proteins could considerably affect the local calcium dynamics.

We thank the reviewer for raising this important point. The reviewer is correct that cytosolic buffers (such as troponin C, ATP, and mobile/fixed endogenous cytosolic buffers) are assumed to be uniformly distributed across all cytosolic domain voxels in our baseline formulation. We fully agree that local variations in cytosolic buffer density near major transport sites (e.g., concentrated troponin C near myofilaments, or localized calmodulin near RyR2 and SERCA complexes) could modulate both local peak free $\text{Ca}^{2+}$ concentrations and diffusion gradients between Z-planes.

In our manuscript, we focused on luminal CASQ2 localization because its specific spatial tethering near RyR2 by triadin and junctin is well established experimentally. However, we agree that non-uniform cytosolic buffering represents an important potential modulator of nanoscale dynamics. To address this, and given its relevance, we have added a full discussion section clarifying how localized cytosolic buffering might influence local source amplitude versus inter-plane transport, and highlighting this as a promising direction for future high-resolution modeling.

6. The discussion occasionally presents CASQ2 co-localization as “solving” the source strength problem required for wave propagation. This wording seems physiologically misleading, since the waves considered here are spontaneous and potentially arrhythmogenic. CASQ2 localization does not solve a requirement of normal excitation; rather, in the model it provides sufficient local source strength for pathological calcium waves to propagate. This distinction should be made clearer.

We thank the reviewer for this comment. We completely agree that the phrasing "solves the source strength problem" could be misinterpreted as implying that facilitating spontaneous waves is a desirable physiological goal.

As the reviewer rightly points out, spontaneous intracellular calcium waves are under large calcium loads, primarily arrhythmogenic, non-physiological phenomena. What CASQ2 co-localization actually provides is the biophysical mechanism that enables local release events to overcome cytosolic attenuation and local SR depletion—thus supplying the local source strength required to sustain inter-plane wave propagation during conditions of calcium overload. Previous models of calcium wave propagation generated calcium waves through a direct link between regions with low calcium buffering, unrealistically connecting subsarcolemmal spaces with very small volumes and low buffer concentrations through diffusion. In doing so, they bypassed the crucial problem of calcium attenuation due to buffering between RyR2 clusters, which are mainly concentrated in the Z-planes.

We have revised the wording in the Discussion to eliminate the misleading phrasing ("solving the problem") and to explicitly clarify that CASQ2–RyR2 co-localization acts as a structural amplifier of local source strength that inadvertently facilitates pathological, arrhythmogenic wave propagation under elevated calcium loads.

7. The discussion could also mention a broader modelling implication of the results. The marked difference between localized and uniformly distributed CASQ2 suggests that spatially averaged representations of proteins may be inadequate when local concentrations near channels or transporters determine the dynamics. More generally, the results caution against replacing heterogeneous protein distributions by uniform effective concentrations in spatially resolved models.

We thank the reviewer for highlighting this important point. We fully agree that the stark divergence in wave dynamics between localized and spatially averaged CASQ2 serves as a general cautionary tale for computational biology: when local microdomain dynamics govern channel gating or release kinetics, substituting a heterogeneous protein architecture with a uniform effective concentration can lead to qualitatively inaccurate predictions.

This links to the response to comment 5 of the referee. This is why we have created a new full section in the discussion that addresses both comments 5 and 7.There we explicitly emphasize this point, framing our findings as a broader methodological caution against using spatially averaged protein distributions in continuum and spatially resolved models of intracellular signaling.

8. Several previous studies have considered spatially resolved modelling of calcium sparks and waves in ventricular myocytes, including the initiation and propagation of spontaneous waves, the role of microscopic dyadic function, and the influence of subcellular geometry and protein localization [4–6]. These studies appear relevant to the present work and should be considered in positioning the model and its contribution relative to the existing literature.

We have extended the introduction to the model in the methodology to acknowledge these previous works.

Minor comments

1. Reference [2] is incomplete.

2. Reference [20] has an incorrect author list; Lederer is not the first author.

Fixed.

3. The cell size is specified as follows: “The cell is represented as a 3D grid of voxels, each measuring 100 nm x 100 nm x 100 nm (see Figure 1). A typical full cell consists of 300 x 1900 x 150 voxels.” This corresponds to a volume of $85.5$ pL, which appears unusually large for a mouse ventricular cardiomyocyte. Please clarify the relation between the rectangular computational domain and the physiological cell volume.

The referee is right here that a typical mice cell is not 85 pl. For mice, the typical volume is ~40-50 pL with a large variability. Some experimental cells are roughly 200 micron long and 30 microns wide, with a depth that is around 10 micron reaching 60 or 70 pl. Meanwhile other cells have more depth, around 15 microns, but are much shorter. So, the values we put as typical are indeed encountered in cells but not normally together. We have clarified now the typical volume of mouse cells and the examples we encounter. A typical large mouse cell is 300 x 1900 x 100 or 57 pL while other typical cells are 250 x 1200 x 150 or 45 pl. To analyze waves we concentrated on those cell that are longer and wide but not very thick. We actually compute typically only a few micron of the depth of the cell (300 x 1900 x 9).

4. I found the statement “with calcium ions diffusing between neighboring voxels in both the SR and the cytosol” somewhat unclear. It may be read as implying that calcium diffuses freely between the SR and cytosol, which would be incorrect. The intended meaning is clear from the equations, but the text should be made unambiguous.

We thank the reviewer for pointing out this potential ambiguity. We agree that the original phrasing could inadvertently be misread as suggesting cross-membrane diffusion between the SR and cytosolic compartments, rather than intra-compartmental diffusion within each respective space.

We have revised the sentence to explicitly clarify that diffusion occurs within each distinct compartment independently (intra-SR diffusion within the luminal grid and intra-cytosolic diffusion within the cytosolic grid), governed strictly by membrane transport and release kinetics across the SR membrane.

5. The statement “O_RyR is the fraction of RyR2 in the clusters that are in the open state” is also somewhat confusing. O_RyR appears to be a local variable representing the open probability of a channel, or equivalently the expected local fraction of open channels. Should equations (4) and (5) be interpreted locally, with separate values in each RyR2-containing voxel, or are the same values

used throughout the cell?

We thank the reviewer for pointing out this potential ambiguity in our definition of $O_{\text{RyR}}$. The reviewer is entirely correct: $O_{\text{RyR}}$ is indeed a local variable calculated independently for each RyR2-containing voxel, representing the local open probability (or expected local fraction of open channels) within that specific sub-micrometric volume based on its local free $\text{Ca}^{2+}$ concentration. It is not a cell-wide global variable.

To eliminate any confusion, we have updated the text in the Methods section accompanying Equations (4) and (5). We explicitly clarify that $O_{\text{RyR}}$ is evaluated locally within each voxel containing RyR2 channels, driven by the local cytosolic and luminal calcium concentrations in that precise grid node.

6. The Data Availability statement indicates that the source code is available only from the corresponding author upon reasonable request. Since the conclusions depend on a substantial computational model, the full source code, parameter files, and instructions required to reproduce the simulations should be made directly available in a public repository.

The data is now available.

------------------------------------------

Reviewer #2: This is an interesting and rigorous study from Conesa and colleagues that demonstrates the importance of calsequestrin colocalization with ryanodine receptors in the facilitation of intracellular calcium waves in cardiomyocytes. The manuscript is well written.

I have a few mostly minor comments for the authors to address.

1. My primary question is that the authors consider only two binary extremes - CASQ only localized with RyR2 or completely uniform. It seems plausible that there is an intermediate regime that likely occurs physiologically - with a majority of CASQ colocalized with RyR2 with the remaining fraction more distributed.

While a full parameter sweep of these scenarios by that fraction is not needed, some consideration of this intermediate scenario is important.

We have considered intermediate values of CASQ2-RyR2 co-localization, showing that, as co-localization diminishes, the waves propagate slower, until at some critical value they cease to propagate and, rather, form domains. This is now shown in the Supporting file S1 where we show the differences in wave propagation and how this propagation breaks down at around 50 % of co-localization. We have added the following text in the main manuscript at the end of the subsection: Uniform CASQ2 distribution abolishes wave propagation.

*Intermediate levels of CASQ2 localization (see Figure S1 in S1 File) result in slower wave propagation and shorter wavelengths as CASQ2 becomes progressively less co-localized with RyR2, until propagation fails when roughly 50 % of CASQ2 is no longer co-localized with RyR2. The case of full co-localization shows very similar wave speed and width to that obtained with approximately 80 % RyR2–CASQ2 co-localization, indicating that the results presented here are robust to physiologically realistic levels of co-localization.*

2. Methods: The authors reference two different voxel counts (specifically in the z-dimension). Please clarify which case is shown for specific results.

We have now clarified this point regarding the typical volume of ventricular mouse cell as encountered in the experiments and the typical sizes we have used in the simulations in the methodology. We clarify that all results presented simulate only a central thin layer of the cell 300 × 1900 × 9 voxels but, internally, we have checked the main results with a larger number of voxels in the Z-dimensions (27 and very specific cases 150) to check that we get the same results.

3. Methods: (Minor) Eqns 1 and 2: The "d" should be written for a PDE, not ODE

We thank the reviewer for pointing out this typo. It is fixed now.

4. Discussion: The authors introduce a critical conceptual point in the manuscript - that the specific spatial organization of specific proteins may be just as important as the total concentrations. Can the authors expand on this in the Discussion, referencing both the importance here as well as other physiological examples, in cardiomyocytes and other fields (as relevant, such as neuroscience)?

We thank the reviewer for this suggestion. We completely agree that our findings highlight a fundamental biophysical principle extending well beyond luminal calcium buffering: namely, that sub-micrometric spatial organization can dictate signaling dynamics and cellular transitions just as strongly as overall protein expression levels. In response, and given that this point has been arisen by multiple referees, we have expanded our Discussion to contextualize this concept across both cardiac electrophysiology and neurobiology.

5. The S2 Github repository was not available. I suggest referencing the vidoes in the repository to the corresponding cases throughout the manuscript.

It is available now.

------------------------------------------------

Reviewer #3: This manuscript presents a 3D model of calcium wave propagation in ventricular myocytes. The main finding is that CASQ2 near RyR2 clusters helps maintain local SR calcium release and supports wave propagation. The study is clear and the main result is interesting. 

Materials and Methods
1. Calcium waves in isolated ventricular mouse： 543nM should be 543nm

Fixed it.

2. Figure 1 shows human atrial cells, while the model represents mouse ventricular myocytes. Please explain how the imaging data were used to define CASQ2 localization in the mouse ventricular model. It would also be helpful to state whether similar localization has been reported in mouse ventricular cells.

We apologize for the error in the caption of figure 1. As described in the methodology all experiments were done in mouse ventricular cells. Figure 1A show co-localization levels in mouse ventricular cells. Originally, we had thought to include different subcellular data for both human and mouse cells but finally we decided to put only data for mouse ventricle and we did not update the figure caption properly. We have now corrected the figure caption.

Results:
1. The transition from failed to sustained propagation occurs between CTOT=170 and 175 μmol/Lcell. Please briefly discuss how sensitive this threshold is to model parameters or RyR2 spatial organization.

We completely agree that the robustness of the propagation threshold is an important issue. Our analysis indicates that small variations in the model parameters do not appreciably affect the transition threshold. We have investigated the effects of changes in SERCA properties and in the parameters governing RyR2 inactivation. These changes can affect the type and structure of the dynamical transition and the resulting wave behavior when the parameter variations become substantial, for example, when the SERCA parameters are more representative of those of rabbit or human cells. However, within a range of small changes in parameter values, the propagation threshold remains largely unchanged.

Thus, a systematic analysis of the robustness of the different transitions to variations in SERCA and RyR2 properties is currently the focus of ongoing work, and we prefer to leave these issues outside the scope of the present manuscript. In the present manuscript, we have therefore chosen to focus on the effect of RyR2-CASQ2 co-localization and leave a comprehensive analysis of SERCA and RyR2 parameter sensitivity for a future publication.

2. The study compares fully localized CASQ2 with a uniform CASQ2 distribution. The authors already note that these are two extreme cases. If possible, including one or more intermediate CASQ2 distributions would help show whether the effect changes gradually or has a clear threshold.

Given that this important point has been raised by different referees we have considered intermediate values of CASQ2-RyR2 co-localization, showing that, as co-localization diminishes, the waves propagate slower, until at some critical value they cease to propagate and, rather, form domains. This is now shown in the Supporting file S1 where we show the differences in wave propagation and how this propagation breaks down at around 50 % of co-localization. We have added the following text in the main manuscript at the end of the subsection: Uniform CASQ2 distribution abolishes wave propagation.

*Intermediate levels of CASQ2 localization (see Figure S1 in S1 File) result in slower wave propagation and shorter wavelengths as CASQ2 becomes progressively less co-localized with RyR2, until propagation fails when roughly 50 % of CASQ2 is no longer co-localized with RyR2. The case of full co-localization shows very similar wave speed and width to that obtained with approximately 80 % RyR2–CASQ2 co-localization, indicating that the results presented here*

*are robust to physiologically realistic levels of co-localization.*

3. The Discussion relates the estimated NCX current to DADs and human AF myocytes. However, the model does not include membrane voltage or other membrane currents. Please explain how NCX flux was converted to current and how increased NCX activity in AF may affect this interpretation. The DAD conclusions should be described as estimates, not direct model results.

The referee is right that the model does not include other membrane currents and operates at a fixed membrane potential. The translation from flux into current was done for purely informative purposes. We have now clarified in the Discussion that the transformation of units from µM/ms (10⁻³ M/s) to pA/pF is done purely for descriptive purposes: we simply divide the flux by the cell's capacitance per volume, C_q (in F/liter), and multiply by Faraday's constant, i.e. I(A/F) = J·F/C_q, using typical values from the literature for this capacitance per volume, which is related to the degree of tabulation typical of the cell type, in order to compare these NCX extrusion values with those found in the literature.

Beyond this methodological caveat, there is a further, and perhaps more important, source of uncertainty: relating the parametrization of NCX used in a spatially resolved model such as ours to the parametrizations typically used in compartmental 0D models or in CRU-based models is not straightforward, and presents uncertainties. For this reason, what matters is not the absolute magnitude of the estimated current but the relative magnitude between waves and global activation.

We have rewritten this part of the discussion to clarify that the core message we wanted to convey is that a wave produces a current roughly ten times lower than that produced by global activation, making global activation associated with substantially greater NCX extrusion. We have also now clarified that these are purely estimates, and that a fully realistic model would be needed to capture the resulting effects on the generation of DADs. As indicated in perspectives, our work opens the possibility to study the propagation of calcium waves within a fully spatially resolved subcellular model where all the currents could be included to analyze DADs properly. More specifically, our model can be used as the core of a full subcellular model that incorporates realistic sodium, potassium, and L-type calcium currents, so that the relative arrhythmogenic relevance of global activation and waves can be properly tested.
