# Supplementary material for: Calsequestrin localization at RyR2 clusters enables calcium wave propagation in ventricular myocytes
Source: PLoS Comput Biol. 2026 Oct 26;22(10):e1014449. doi: 10.1371/journal.pcbi.1014449 (PMC13754233; doi:10.1371/journal.pcbi.1014449)
Supplement: Peer review history

# Peer review: Decision Letter 0

**DOI:** 10.1371/journal.pcbi.1014449.r001

**Authors:** Gary R Mirams, Boyce Griffith

14 Jul 2026

Calsequestrin localization at RyR2 clusters enables calcium wave propagation in ventricular myocytes

PLOS Computational Biology

Dear Dr. Alvarez-Lacalle,

Thank you for submitting your manuscript to PLOS Computational Biology. After careful consideration, we feel that it has merit but does not fully meet PLOS Computational Biology's publication criteria as it currently stands. Therefore, we invite you to submit a revised version of the manuscript that addresses the points raised during the review process.

Please submit your revised manuscript by Sep 12 2026 11:59PM. If you will need more time than this to complete your revisions, please reply to this message or contact the journal office at ploscompbiol@plos.org. When you're ready to submit your revision, log on to https://www.editorialmanager.com/pcompbiol/ and select the 'Submissions Needing Revision' folder to locate your manuscript file.

Please include the following items when submitting your revised manuscript:

* A letter that responds to each point raised by the editor and reviewer(s). You should upload this letter as a separate file labeled 'Response to Reviewers'. This file does not need to include responses to formatting updates and technical items listed in the 'Journal Requirements' section below.

* A marked-up copy of your manuscript that highlights changes made to the original version. You should upload this as a separate file labeled 'Revised Manuscript with Track Changes'.

* An unmarked version of your revised paper without tracked changes. You should upload this as a separate file labeled 'Manuscript'.

Kind regards,

Gary R Mirams, Ph.D.

Academic Editor

PLOS Computational Biology

Boyce Griffith

Section Editor

PLOS Computational Biology

**Additional Editor Comments (if provided):**

We welcome a revision based upon addressing the reviewer comments, please note that for Reviewer 1 these are in a separate PDF file.

Please note the PLoS CB code sharing requirements: https://journals.plos.org/ploscompbiol/s/code-availability so if possible make the Github repository public on re-submission, or provide a zip file of its contents for review.

**Journal Requirements:**

If the reviewer comments include a recommendation to cite specific previously published works, please review and evaluate these publications to determine whether they are relevant and should be cited. There is no requirement to cite these works unless the editor has indicated otherwise.

1) Please ensure that the CRediT author contributions listed for every co-author are completed accurately and in full.

At this stage, the following Authors/Authors require contributions: David Conesa, Blas Echebarria, Leif Hove-Madsen, Yohannes Shiferaw, and Enrique Alvarez-Lacalle. Please ensure that the full contributions of each author are acknowledged in the "Add/Edit/Remove Authors" section of our submission form.

The list of CRediT author contributions may be found here: https://journals.plos.org/ploscompbiol/s/authorship#loc-author-contributions

2) We ask that a manuscript source file is provided at Revision. Please upload your manuscript file as a .doc, .docx, .rtf or .tex. If you are providing a .tex file, please upload it under the item type u2018LaTeX Source Fileu2019 and leave your .pdf version as the item type u2018Manuscriptu2019.

3) We noticed that you used the phrase 'not shown' in the manuscript. We do not allow these references, as the PLOS data access policy requires that all data be either published with the manuscript or made available in a publicly accessible database. Please amend the supplementary material to include the referenced data or remove the references.

4)Thank you for stating "The minimal data set supporting the conclusions of this study is available at https://github.com/ConesaJR/CASQ2_RyR2_waves/. The repository contains videos illustrating calcium-wave propagation. The source code used in this study is available from the corresponding author upon reasonable request.." However, we were unable to access the data using the information provided. Please review and update the Data Availability Statement to ensure that the underlying data are publicly accessible and that all accession numbers, links, or repository details are correct and functional.

**Reviewers' comments:**

Reviewer's Responses to Questions

**Comments to the Authors:**

**Please note here if the review is uploaded as an attachment.**

Reviewer #1: See enclosed report

Reviewer #2: This is an interesting and rigorous study from Conesa and colleagues that demonstrates the importance of calsequestrin colocalization with ryanodine receptors in the facilitation of intracellular calcium waves in cardiomyocytes. The manuscript is well written.

I have a few mostly minor comments for the authors to address.

1. My primary question is that the authors consider only two binary extremes - CASQ only localized with RyR2 or completely uniform. It seems plausible that there is an intermediate regime that likely occurs physiologically - with a majority of CASQ colocalized with RyR2 with the remaining fraction more distributed.

While a full parameter sweep of these scenarios by that fraction is not needed, some consideration of this intermediate scenario is important.

2. Methods: The authors reference two different voxel counts (specifically in the z-dimension). Please clarify which case is shown for specific results.

3. Methods: (Minor) Eqns 1 and 2: The "d" should be written for a PDE, not ODE

4. Discussion: The authors introduce a critical conceptual point in the manuscript - that the specific spatial organization of specific proteins may be just as important as the total concentrations. Can the authors expand on this in the Discussion, referencing both the importance here as well as other physiological examples, in cardiomyocytes and other fields (as relevant, such as neuroscience)?

5. The S2 Github repository was not available. I suggest referencing the vidoes in the repository to the corresponding cases throughout the manuscript.

Reviewer #3: This manuscript presents a 3D model of calcium wave propagation in ventricular myocytes. The main finding is that CASQ2 near RyR2 clusters helps maintain local SR calcium release and supports wave propagation. The study is clear and the main result is interesting.

Materials and Methods

1. Calcium waves in isolated ventricular mouse 543nM should be 543nm

2. Figure 1 shows human atrial cells, while the model represents mouse ventricular myocytes. Please explain how the imaging data were used to define CASQ2 localization in the mouse ventricular model. It would also be helpful to state whether similar localization has been reported in mouse ventricular cells.

Results:

1. The transition from failed to sustained propagation occurs between CTOT=170 and 175 μmol/Lcell. Please briefly discuss how sensitive this threshold is to model parameters or RyR2 spatial organization.

2. The study compares fully localized CASQ2 with a uniform CASQ2 distribution. The authors already note that these are two extreme cases. If possible, including one or more intermediate CASQ2 distributions would help show whether the effect changes gradually or has a clear threshold.

3. The Discussion relates the estimated NCX current to DADs and human AF myocytes. However, the model does not include membrane voltage or other membrane currents. Please explain how NCX flux was converted to current and how increased NCX activity in AF may affect this interpretation. The DAD conclusions should be described as estimates, not direct model results.

**********

**Have the authors made all data and (if applicable) computational code underlying the findings in their manuscript fully available?**

The PLOS Data policy requires authors to make all data and code underlying the findings described in their manuscript fully available without restriction, with rare exception (please refer to the Data Availability Statement in the manuscript PDF file). The data and code should be provided as part of the manuscript or its supporting information, or deposited to a public repository. For example, in addition to summary statistics, the data points behind means, medians and variance measures should be available. If there are restrictions on publicly sharing data or code —e.g. participant privacy or use of data from a third party—those must be specified.

Reviewer #1: **No:**  The code is not publically available; see report.

Reviewer #2: **No:**  Authors reference the code being in S2 Github repository, but at time of review, this was not available.

Reviewer #3: Yes

**********

PLOS authors have the option to publish the peer review history of their article (what does this mean?). If published, this will include your full peer review and any attached files.

If you choose “no”, your identity will remain anonymous but your review may still be made public.

**Do you want your identity to be public for this peer review?**  For information about this choice, including consent withdrawal, please see our Privacy Policy.

Reviewer #1: **Yes:**  Aslak Tveito

Reviewer #2: No

Reviewer #3: No

[NOTE: If reviewer comments were submitted as an attachment file, they will be attached to this email and accessible via the submission site. Please log into your account, locate the manuscript record, and check for the action link "View Attachments". If this link does not appear, there are no attachment files.]

**Figure resubmission:**

**Reproducibility:**

?>

Attachment Submitted filename: Report.pdf

# Author comment: Author response to Decision Letter 1

**DOI:** 10.1371/journal.pcbi.1014449.r002

11 Sep 2026

Attachment Submitted filename: Response_referees_CASQ2_PLOSCB.docx

# Peer review: Decision Letter 1

**DOI:** 10.1371/journal.pcbi.1014449.r003

**Authors:** Gary R Mirams, Boyce Griffith

28 Sep 2026

Dear Dr. Alvarez-Lacalle,

We are pleased to inform you that your manuscript 'Calsequestrin localization at RyR2 clusters enables calcium wave propagation in ventricular myocytes' has been provisionally accepted for publication in PLOS Computational Biology.

Before your manuscript can be formally accepted you will need to complete some formatting changes, which you will receive in a follow up email. A member of our team will be in touch with a set of requests.

Please note that your manuscript will not be scheduled for publication until you have made the required changes, so a swift response is appreciated.

IMPORTANT: The editorial review process is now complete. PLOS will only permit corrections to spelling, formatting or significant scientific errors from this point onwards. Requests for major changes, or any which affect the scientific understanding of your work, will cause delays to the publication date of your manuscript.

Should you, your institution's press office or the journal office choose to press release your paper, you will automatically be opted out of early publication. We ask that you notify us now if you or your institution is planning to press release the article. All press must be co-ordinated with PLOS.

Thank you again for supporting Open Access publishing; we are looking forward to publishing your work in PLOS Computational Biology.

Best regards,

Gary R Mirams, Ph.D.

Academic Editor

PLOS Computational Biology

Boyce Griffith

Section Editor

PLOS Computational Biology

***********************************************************

Many thanks for a thorough revision

Reviewer's Responses to Questions

**Comments to the Authors:**

**Please note here if the review is uploaded as an attachment.**

Reviewer #1: The authors have adequately addressed all of my comments, and I have no further concerns.

Reviewer #2: The authors have addressed all of my concerns.

Reviewer #3: Thank you for addressing my previous comments. The manuscript has been improved and I have no further comments.

**********

**Have the authors made all data and (if applicable) computational code underlying the findings in their manuscript fully available?**

The PLOS Data policy requires authors to make all data and code underlying the findings described in their manuscript fully available without restriction, with rare exception (please refer to the Data Availability Statement in the manuscript PDF file). The data and code should be provided as part of the manuscript or its supporting information, or deposited to a public repository. For example, in addition to summary statistics, the data points behind means, medians and variance measures should be available. If there are restrictions on publicly sharing data or code —e.g. participant privacy or use of data from a third party—those must be specified.

Reviewer #1: Yes

Reviewer #2: Yes

Reviewer #3: None

**********

PLOS authors have the option to publish the peer review history of their article (what does this mean?). If published, this will include your full peer review and any attached files.

If you choose “no”, your identity will remain anonymous but your review may still be made public.

**Do you want your identity to be public for this peer review?**  For information about this choice, including consent withdrawal, please see our Privacy Policy.

Reviewer #1: **Yes:**  Aslak Tveito

Reviewer #2: **Yes:**  Seth Weinberg

Reviewer #3: No

# Editor report: Acceptance letter

**DOI:** 10.1371/journal.pcbi.1014449.r004

**Authors:** Gary R Mirams, Boyce Griffith

PCOMPBIOL-D-26-01438R1

Calsequestrin localization at RyR2 clusters enables calcium wave propagation in ventricular myocytes

Dear Dr Alvarez-Lacalle,

I am pleased to inform you that your manuscript has been formally accepted for publication in PLOS Computational Biology. Your manuscript is now with our production department and you will be notified of the publication date in due course.

The corresponding author will soon be receiving a typeset proof for review, to ensure errors have not been introduced during production. Please review the PDF proof of your manuscript carefully, as this is the last chance to correct any errors. Please note that major changes, or those which affect the scientific understanding of the work, will likely cause delays to the publication date of your manuscript.

Soon after your final files are uploaded, unless you have opted out, the early version of your manuscript will be published online. The date of the early version will be your article's publication date. The final article will be published to the same URL, and all versions of the paper will be accessible to readers.

For Research, Software, and Methods articles, you will receive an invoice from PLOS for your publication fee after your manuscript has reached the completed accept phase. If you receive an email requesting payment before acceptance or for any other service, this may be a phishing scheme. Learn how to identify phishing emails and protect your accounts at https://explore.plos.org/phishing.

Thank you again for supporting PLOS Computational Biology and open-access publishing. We are looking forward to publishing your work!

With kind regards,

Janani Seenivasan

PLOS Computational Biology | Carlyle House, Carlyle Road, Cambridge CB4 3DN | United Kingdom ploscompbiol@plos.org | Phone +44 (0) 1223-442824 | ploscompbiol.org | @PLOSCompBiol
